# Clinical Characteristics of Turkish Women with *Candida krusei* Vaginitis and Antifungal Susceptibility of the *C. krusei* Isolates 

**DOI:** 10.1155/2013/698736

**Published:** 2013-12-11

**Authors:** Ahmet Bariş Güzel, Merve Aydın, Melda Meral, Ayşe Kalkancı, Macit Ilkit

**Affiliations:** ^1^Department of Obstetrics and Gynecology, Faculty of Medicine, University of Çukurova, 01330 Adana, Turkey; ^2^Department of Microbiology, Faculty of Medicine, University of Gazi, 06500 Ankara, Turkey; ^3^Department of Microbiology, Faculty of Medicine, University of Erzincan, 24000 Erzincan, Turkey; ^4^Division of Mycology, Department of Microbiology, Faculty of Medicine, University of Çukurova, 01330 Adana, Turkey

## Abstract

*Objective*. *Candida krusei* causes approximately 1% of vulvovaginal candidiasis (VVC) cases and is naturally resistant to fluconazole. Antifungal testing may be required if *C. krusei* vaginitis fails to respond to non-fluconazole therapy, particularly in patients with recurrent infections. *Design*. We investigated the clinical characteristics and antifungal susceptibility profile of vaginal *C. krusei* isolates. Between 2009 and 2012, we identified 560 unrelated *Candida* spp.-positive vaginal cultures, of which 28 (5.0%) were *C. krusei*. These isolates were analyzed according to host factors and the clinical forms of VVC, and their *in vitro* susceptibility to 10 antifungal agents was tested using a reference microdilution method. *Results*. We observed that perineal laceration and increased age (>50 years) were significant predictors of *C. krusei* in vaginal samples (*P* < 0.05). All isolates were susceptible to amphotericin B, caspofungin, ketoconazole, and miconazole. Additionally, susceptible dose-dependent and resistant rates were found for fluconazole as 42.9% and 57.1%, respectively. Remarkably, only 42.9% and 67.9% of the isolates were susceptible to itraconazole and voriconazole, respectively. *Conclusions*. Understanding local susceptibility patterns, especially those of non-*C. albicans Candida* species, can significantly aid in the selection of an effective antifungal agent. The *in vivo* response of *C. krusei* vaginitis to various antifungal therapeutics remains unknown and requires further research.

## 1. Introduction

Vulvovaginal candidiasis (VVC) is a common illness attributed to an overgrowth of *Candida* species, and it is estimated that 75% of all women will experience an episode of VVC in their lifetimes. *C*. *albicans* accounts for 80–95% of all episodes of VVC worldwide [1, 2]. The prevalence of VVC due to non-*C*. *albicans Candida* species previously ranged from 5 to 20%; however, the number of reported cases has increased sharply over the last two decades, particularly for cases of *C*. *glabrata *[3, 4]. Therefore, the possibility of antifungal resistant strains of non-*C*. *albicans Candida* species in *Candida *vaginitis should be considered in clinics. The emergence of resistance may be attributed to the following factors (i) the widespread use of over-the-counter (OTC) medications; (ii) long-term use of suppressive azoles; and (iii) the frequent use of courses of antifungal medications [1, 3] or (iv) the increase use of vaginal cultures for reliable diagnoses [2, 5]. There is no evidence to suggest the followings: (i) certain women may be more susceptible to infection by particular *Candida *species over other species, or (ii) there are epidemiologic factors that may predispose women to acute VVC (AVVC) versus recurrent VVC (RVVC) [1, 2].

VVC is also, albeit infrequently, caused by *C. parapsilosis*, *C*. *tropicalis*, and *C*. *krusei *[1, 6, 7]. The decreased susceptibility of bloodstream *C*. *krusei *isolates to amphotericin B and 5-flucytosine as determined using the broth microdilution method is well documented [8]. However, *in vitro* susceptibility testing has not been used to evaluate the clinical response of *C*. *krusei *vaginitis [9]. In addition, little is known about vaginal *C*. *krusei* infections because they are relatively rare. However, *C*. *krusei* is known to be inherently resistant to one of the most commonly used antifungal drugs, fluconazole. The signs and symptoms of *C*. *krusei* vaginitis appear to be indistinguishable from the signs and symptoms of VVC cases caused by other *Candida *species [6, 10]. Although rare, *C*. *krusei* is an intractable cause of RVVC. Furthermore, most institutions have had limited experience with *C*. *krusei *vaginitis [6]. Thus, the present study aims to fill this gap in the literature. Here, we retrospectively analyzed the epidemiological characteristics of 28 vaginal *C*. *krusei *isolates, including host and risk factors. In addition, we investigated the antifungal susceptibility profiles of these isolates to 10 antifungal drugs to determine the most appropriate therapeutic choice(s) in women with *C*. *krusei *vaginitis.

## 2. Materials and Methods

### 2.1. Vaginal *C. krusei* Isolates

We examined 1,543 vaginal samples from unrelated women, of which 560 (36.3%) were culture-positive and 983 (63.7%) were culture-negative for *Candida* yeasts, and the medical records of these cases were reviewed. Among the 560 vaginal yeast isolates, *C*. *albicans *was the most common species and identified in 242 (43.2%) isolates, followed by *C*. *glabrata* in 155 (27.7%), *C*. *krusei* in 28 (5.0%), *C*. *kefyr* in 20 (3.6%), and in 115 (20.5%) representing several species of *Candida*. Women who had *C*. *krusei *in their vagina were included in the study. The definitions of the clinical presentations of VVC for each group were as follows: AVVC (group 1), currently asymptomatic women with initial or sporadic episodes of symptomatic vaginitis, that is, occurring fewer than four times per year (*n* = 8); RVVC (group 2), symptomatic patients with a history of four or more clinical episodes of VVC per year (*n* = 13); and controls (group 3), women who incidentally carried a normal level of *C. krusei* in their vaginal culture without vaginitis, who were completely asymptomatic and had no history of RVVC (*n* = 7). The control group included a mixed group of asymptomatic women who had no history of RVVC and women who had positive cultures. All participants took part in a short interview, which included questions regarding lifestyle and medical, gynecological, and sexual history. This study was reviewed and approved by the Institutional Review Board at the University of Çukurova, Adana, Turkey. The Declaration of Helsinki protocols were followed, and the patients provided written informed consent.

### 2.2. Identification of *C. krusei*

The *C. krusei* isolates were recovered on CHROMagar Candida (Becton Dickinson, Heidelberg, Germany) and appeared as dull, flat, light mauve to mauve, and colonies with a whitish border. The criteria for the identification of *C*. *krusei* were the absence of germ tube production in human serum at 37°C at 2 hours, the production of abundant pseudohyphae with some moderate branching on cornmeal-Tween 80 agar (Difco, Detroit, MI, USA), and weak or absent urease activity. These isolates were verified by their assimilation patterns using the API 20C AUX method (bioMérieux, Marcy l'Étoile, France) [11].* C*. *krusei* ATCC 6258 was used as a positive control.

### 2.3. Antifungal Susceptibility Testing

Antifungal testing was conducted at the Department of Microbiology, Faculty of Medicine, Gazi University, Ankara, using a broth microdilution method and according to the guidelines of the M27-A3 document of the Clinical and Laboratory Standards Institute (CLSI). Before testing, each isolate was subcultured on Sabouraud glucose agar (SGA; Merck, Darmstadt, Germany) to ensure purity and viability. The interpretation of antifungal susceptibility was guided by criteria derived from the CLSI's M27-A3 protocol [12]. The following antifungal agents were tested: amphotericin B (0.03–16 *μ*g/mL), 5-flucytosine (0.06–64 *μ*g/mL), caspofungin (0.03–16 *μ*g/mL), fluconazole (0.12–128 *μ*g/mL), itraconazole (0.03–16 *μ*g/mL), voriconazole (0.008–16 *μ*g/mL), econazole (0.007–8 *μ*g/mL), ketoconazole (0.007–8 *μ*g/mL), miconazole (0.007–8 *μ*g/mL), and sulconazole (0.03–16 *μ*g/mL).

The minimal inhibitory concentrations (MICs) were determined for each antifungal agent and used to classify the susceptibility of the isolates as follows: (i) amphotericin B, MIC ≤ 1 (*μ*g/mL), susceptible (S); (ii) 5-flucytosine, MIC ≤ 4 (*μ*g/mL) S, MIC between 8 and 16 (*μ*g/mL) intermediate (I), MIC ≥ 32 (*μ*g/mL) resistant (*R*); (iii) caspofungin, MIC ≥ 2 (*μ*g/mL) *R*; (iv) fluconazole, MIC ≤ 8 (*μ*g/mL) S, MIC between 16 and 32 (*μ*g/mL) susceptible dose dependent (S-DD), MIC ≥ 64 (*μ*g/mL) *R*; (v) itraconazole, MIC ≤ 0.125 (*μ*g/mL) S, MIC between 0.25 and 0.5 (*μ*g/mL) S-DD, MIC ≥ 1 (*μ*g/mL) R; (vi) voriconazole, ≤ 1 (*μ*g/mL) S, MIC = 2 (*μ*g/mL) S-DD, MIC ≥ 4 (*μ*g/mL) *R*; (vii) ketoconazole, MIC ≥ 16 (*μ*g/mL) *R*; and (viii) miconazole, MIC ≥ 4 (*μ*g/mL) *R* [12]. Currently, there are no published criteria for defining econazole and sulconazole susceptibility [13]. These results were expressed in terms of the MIC range and the MIC_50_, and MIC_90_ values for each antifungal agent. All *C*. *krusei* isolates were declared resistant to fluconazole. *C*.* krusei* ATCC 6258 and *C*. *parapsilosis *ATCC 22019 were used as controls, as recommended by the CLSI [12, 14].

### 2.4. Statistical Analysis

Data were analyzed using IBM SPSS version 19. Continuous variables, such as age and body mass index, were first divided into bins: <30, 30–39, 40–49, and >50 years of age and <25 (under or normal weight) and >25 (overweight or obese) for body mass index. Then, all categorical variables were cross classified by *C*. *krusei* infection or carrier status to descriptively summarize the association between the variables using the chi-squared test and to measure the degree of association using the odds ratio with a 95% confidence interval. For the multivariate analysis, logistic regression modeling of the binary data (*C*. *krusei* infected, carrier, or neither) was used to determine the significant predictors of *C*. *krusei *infection, after adjusting for other factors in the models. Factors with significance levels <0.30 were entered into the multivariate logistic model to determine the significant effect of each factor simultaneously on the prediction of *C*. *krusei *infection. None of the other factors contributed significantly to the prediction of *C*. *krusei *infection.

## 3. Results

*C*. *krusei* isolates were recovered from non pregnant patients without diabetes mellitus (*n* = 9), pregnant patients (*n* = 6), diabetes mellitus patients (*n* = 6), and contraceptive user's (*n* = 7) with no previous history of immunodeficiency who visited the Faculty of Medicine Department of Obstetrics and Gynecology at Çukurova University from 2009 until 2012. Of the *C*. *krusei* isolates, 24 (85.7%) were the only species on plates and four (14.3%) were part of mixed cultures, which were always included by *C*. *albicans*. In our group, 24 (85.7%) women were in premenopausal and four (14.3%) in postmenopausal period who had also exposed hormone replacement therapy. The mean age of the women was 40.3 ± 10.4 years (range, 21 to 59 years old).

The basic demographic and clinical characteristics of women with vaginal *C*. *krusei *isolates are presented in Tables 1 and 2. Perineal laceration is significantly higher (*P* = 0.006) in the *C*. *krusei* group compared with the non-*C*. *krusei* group (Table 2). As revealed by multivariate analysis, existence of perineal laceration (*P* = 0.009) and an age of over 50 years (*P* = 0.02) were significant predictors of *C*. *krusei *vaginitis or carrier status (Table 3).

The MIC results for the control strains of *C*.* krusei* ATCC 6258 and *C*. *parapsilosis *ATCC 22019 were within the acceptable range. All the *C*. *krusei* isolates were susceptible to amphotericin B, caspofungin, ketoconazole, and miconazole, and 10 of the 28 isolates (35.7%) were defined as S-DD for 5-flucytosine. High MIC rates were observed for fluconazole, of which 42.9% of the isolates were S-DD and 57.1% were *R*. Remarkably, only 42.9% and 67.9% of the isolates were susceptible to itraconazole (six S-DD and 10 *R*) and voriconazole (four S-DD and five *R*), respectively. We also observed low MIC levels for econazole and sulconazole. The antifungal susceptibilities of *C*.* krusei *isolated from patients with AVVC and RVVC did not differ significantly from those isolated from the group of women without symptoms of vaginitis (*P* > 0.05; Table 4).

## 4. Discussion

To the best of our knowledge, this study is the largest series to exclusively investigate the prevalence of, host and risk factors for, and antifungal susceptibility of the minor isolate *C*. *krusei*. We also briefly summarized the baseline and demographic characteristics of women who had *C*. *krusei *present in their vaginal samples (Tables 1 and 2). Our data suggest that the prevalence of *C*. *krusei* is relatively high (5.0%) in this population, displayed no specific host preferences, and was most often associated with RVVC. Perineal laceration and increased age (>50 years) were significant predictors of *C*. *krusei *vaginitis (Tables 2 and 3). An important limitation of the present study is the lack of data regarding *in vivo *therapeutic drug choices and outcomes in *C*. *krusei *vaginitis. In addition, the number of women with *C*. *krusei* is very small, so, for some of the other factors we examined, the study may not have had sufficient power to detect a difference (Tables 1–3).

The presence of mixed cultures may affect the choice of treatment strategy. In our previous study, using chromogenic media, we determined that the percentage of mixed cultures recovered from vaginal samples was as high as 14.1% in Adana, Turkey [4]. The results of this investigation (14.3%) are similar to those of our earlier study. In addition, our finding that older women (mean age, 40.3 years) are more susceptible to infection corroborates the earlier finding of Singh et al. [6] (mean age, 44 years). However, the women studied by Singh et al. [6] all had RVVC, whereas our study included not only RVVC cases but also AVVC and controls. These authors noted that *C*. *krusei* isolates were highly resistant to fluconazole (MIC_90_ > 64 *μ*g/mL), consistent with our findings. In addition, these authors reported resistance to miconazole (MIC_90_ > 4 *μ*g/mL), one of the most commonly used OTC antifungal agents, which we did not observe. However, in line with our results, clotrimazole was observed to be the most active topical imidazole against *C*. *krusei*. Moreover and more importantly, amphotericin B, caspofungin, itraconazole, and voriconazole were demonstrated to have favorable antifungal activity, although, in our study, several strains exhibited obvious high resistance to the latter two drugs. Of note, the authors suggested that the therapy should continue for 2–6 weeks, regardless of the agent used [6]. On the other hand, a recent study reported that fluconazole-resistant *C*. *albicans* appears to be emerging in clinics [15]. Therefore, antifungal susceptibility testing may assist in selecting the appropriate therapeutic drug not only for non-*C*. *albicans Candida *vaginitis but also for rare fluconazole-resistant *C*.* albicans *vaginitis.

In this investigation, amphotericin B, caspofungin, ketoconazole, and miconazole were observed to be active against all *C*. *krusei *isolates (Table 4). In contrast to the findings of Singh et al. [6], but in line with those of Richter et al. [16], itraconazole exhibited high S-DD and *R* rates, 35.7% and 21.4%, respectively. Although the new broad-spectrum oral antifungal voriconazole is rarely used in patients with VVC, we observed that 67.9% of the isolates were susceptible to voriconazole. Pfaller et al. [8] reported a higher rate, stating that 81.5% of 426 genital *C*. *krusei *isolates were susceptible to voriconazole using the CLSI M44-A disk diffusion method. In conrast to our findings, Lyon et al. [17] reported that fluconazole resistance rates were highly predictive of resistance to voriconazole. Although specific clinical cutoff points have not yet been assigned for econazole and sulconazole susceptibility, we observed low MIC values for both drugs. Nystatin suppositories and boric acid could be therapies of choice for *C*. *krusei* vaginitis [6].

This study is the largest to date to investigate the antifungal drug-resistance profile of *C*. *krusei *vaginal isolates and the epidemiologic risk factors of infection. In this investigation, perineal laceration and increasing age (>50 years) were important predictive factors for *C*. *krusei* vaginitis or carrier status (Table 3). This study also revealed that the topical imidazoles (ketoconazole and miconazole), which can be prescribed safely in routine practice, were effective against all *C*. *krusei* isolates. In addition, the vaginal *C*. *krusei* isolates were less susceptible to itraconazole (42.9%) and voriconazole (67.9%) than to other antifungal therapeutics. These findings may have implications for the *in vivo* therapeutic treatment of *C*. *krusei *vaginitis (Table 4). Thus, the identification of *C*. *krusei *in vaginal samples and *in vitro *antifungal testing will assist in the selection of appropriate antifungal agents and therapy duration. Future clinical trials to determine the *in vivo* efficacy of the current drugs for women with *C*. *krusei* vaginitis are required.

## Figures and Tables

**Table 1:** Basic demographic characteristics of women with vaginal complaints in this study.

Variables	*C*. *krusei n* (%)			*P*
	Present	Absent	Total	
Education status				
Illiterate	4 (1.7)	226 (98.3)	230	0.96
Primary school	14 (1.9)	709 (98.1)	723	
Secondary school	2 (1.7)	119 (98.3)	121	
High school	5 (1.5)	338 (98.5)	343	
College	3 (2.4)	122 (97.6)	125	
Marital status				
Single	0 (0.0)	17 (100.0)	17	0.77
Married	26 (1.8)	1,418 (98.2)	1,444	
Widowed-Divorced	2 (2.5)	79 (97.5)	81	
Tobacco use				
Yes	6 (1.8)	328 (98.2)	334	0.6
No	22 (1.8)	1,185 (98.2)	1,207	
Alcohol use				
Yes	0 (0.0)	35 (100.0)	35	0.52
No	28 (1.9)	1,479 (98.1)	1,507	

	Mean ± Standard Deviation			
Age	40.3 ± 10.4	35.3 ± 10.8	35.4 ± 10.8	**0.01**
Gravida	3.4 ± 1.9	2.9 ± 2.1	2.9 ± 2.0	0.2

**Table 2:** Basic clinical characteristics of women with vaginal complaints in the study.

Variables	*C*. *krusei n* (%)			*P*
	Present	Absent	Total	
Diabetes mellitus				
Absent	21 (1.7)	1,250 (98.3)	1,271	0.21
Present	7 (2.6)	265 (97.4)	272	
Hypothyroidism				
Absent	26 (1.8)	1,452 (98.2)	1,478	0.31
Present	2 (3.2)	60 (96.8)	62	
Hyperthyroidism				
Absent	26 (1.7)	1,478 (98.3)	1,504	0.15
Present	2 (5.3)	36 (94.7)	38	
Other chronic diseases				
Absent	19 (1.7)	1,127 (98.3)	1,146	0.28
Present	9 (2.3)	387 (97.7)	396	
Medication other than antibiotics				
Absent	22 (2.0)	1,056 (98.0)	1,078	0.22
Present	6 (1.3)	458 (98.7)	464	
Use of local steroid in the last 4 weeks				
Absent	27 (1.8)	1,492 (98.2)	1,519	0.35
Present	1 (4.3)	22 (95.7)	23	
Use of systemic steroid in the last 4 weeks				
Absent	28 (1.9)	1,482 (98.1)	1,510	0.57
Present	0 (0.0)	30 (100.0)	30	
Perineal laceration				
Absent	15 (1.3)	1,163 (98.7)	1,178	**0.006**
Present	13 (3.6)	351 (96.4)	364	
Contraception				
None	9 (1.4)	637 (98.6)	646	0.49
OC	1 (1.0)	103 (99.0)	104	
IUD	8 (2.9)	268 (97.1)	276	
Condom	4 (1.5)	258 (98.5)	262	
Others	6 (2.4)	248 (97.6)	254	
Personal allergic history				
Absent	24 (1.8)	1,316 (98.2)	1,340	0.51
Present	4 (2.0)	197 (98.0)	201	
History of sexual intercourse in the last 4 weeks				
Present	8 (2.8)	279 (97.2)	287	0.13
Absent	20 (1.6)	1,235 (98.4)	1,255	
Antibiotic use in the last 4 weeks				
Absent	27 (2.1)	1,260 (97.9)	1,287	0.07
Present	1 (0.4)	254 (99.6)	255	
Body mass index				
≤19	1 (2.3)	42 (97.7)	43	0.4
19–24	4 (1.1)	369 (98.9)	373	
24–29	9 (1.6)	561 (98.4)	570	
>29	14 (2.5)	540 (97.5)	554	

OC: oral contraceptive; IUD: intrauterine device.

**Table 3:** Analysis of predictive factors for *Candida  krusei *infection using univariate and multivariate logistic analyses.

Predictores	Univariate analysis			Multivariate analysis		
	OR	*P*	%95 CI	OR	*P*	%95 CI
Age						
<30	1.0	—	—	—	—	—
30–39	2.56	0.09	0.87–7.55	2.4	0.12	0.8–7.16
40–49	2.48	0.14	0.75–8	2.7	0.11	0.79–9.3
≥50	5.39	**0.004**	1.69–17.2	7.9	**0.02**	1.34–46.7
Body mass index	1.72	0.19	0.65–4.57	1.28	0.63	0.46–3.57
Diabetes mellitus	1.57	0.21	0.66–3.74	0.26	0.11	0.05–1.35
Hyperthyroidism	3.2	0.15	0.72–13.8	3.92	0.08	0.84–18.3
Medication other than antibiotics	0.63	0.22	0.25–1.56	0.56	0.23	0.21–1.46
Use of local steroid	2.5	0.35	0.32–19.1	3.11	0.3	0.38–26.2
Perineal laceration	2.87	0.06	1.25–6.1	3.25	**0.009**	1.34–7.87
Antibiotics (last 4-weeks)	0.18	0.07	0.025–1.3	0.19	0.11	0.03–1.48

**Table 4:** Antifungal susceptibility of 28 vaginal *Candida  krusei* isolates stratified according to clinical forms.

Antifungals	Acute VVC (*n* = 7)	Recurrent VVC (*n* = 13)	Control (*n* = 8)
Amphotericin B (*μ*g/mL)			
MIC range	0.03–0.25	0.03–0.5	0.06–0.5
MIC_50_	0.03	0.25	0.25
MIC_90_	0.25	0.25	0.25
R, ≥2 *μ*g/mL			
*n* (%)	—	—	—
5-Flucytosine (*μ*g/mL)			
MIC range	0.125–8	0.125–8	0.125–16
MIC_50_	2	2	8
MIC_90_	8	8	16
R, ≥32 *μ*g/mL			
*n* (%)	—	—	—
Caspofungin (*μ*g/mL)			
MIC range	0.03–0.06	0.03–0.06	0.03–0.06
MIC_50_	0.03	0.03	0.03
MIC_90_	0.06	0.03	0.06
*R*, ≥2 *μ*g/mL			
*n* (%)	—	—	—
Fluconazole (*μ*g/mL)^#^			
MIC range	16–128	16–128	16–128
MIC_50_	128	64	32
MIC_90_	128	128	128
*R*, ≥64 *μ*g/mL			
*n* (%)	4	9	3
S-DD, 16–32 *μ*g/mL			
*n* (%)	3	4	5
Itraconazole (*μ*g/mL)			
MIC range	0.125–16	0.125–16	0.125–8
MIC_50_	0.25	0.25	0.125
MIC_90_	4	4	2
*R*, ≥1 *μ*g/mL			
*n* (%)	3	4	3
Voriconazole (μg/mL)			
MIC range	0.125–8	0.125–16	0.25–8
MIC_50_	1	0.5	0.5
MIC_90_	4	4	2
*R*, ≥4 *μ*g/mL			
*n* (%)	2	2	1
Ketoconazole (*μ*g/mL)			
MIC range	0.125–8	0.25–8	0.25–8
MIC_50_	0.25	4	0.25
MIC_90_	2	8	8
*R*, ≥16 *μ*g/mL			
*n* (%)	—	—	—
Econazole (*μ*g/mL)			
MIC range	0.5–1	0.125–2	0.5–1
MIC_50_	1	1	1
MIC_90_	1	2	1
*R*, ND			
*n* (%)	—	—	—
Miconazole (*μ*g/mL)			
MIC range	0.25–0.5	0.06–1	0.25–0.5
MIC_50_	0.25	0.25	0.25
MIC_90_	0.5	1	0.5
*R*, ≥4 *μ*g/mL			
*n* (%)	—	—	—
Sulconazole (*μ*g/mL)			
MIC range	1–4	0.06–4	1-2
MIC_50_	2	1	1
MIC_90_	4	4	2
*R*, ND			
*n* (%)	—	—	—

VVC: vulvovaginal candidiasis; *R*: resistance; S-DD: susceptible dose dependent; ND: not determined. ^#^All isolates were declared resistant to fluconazole.
